# Senior Housing as a Living Environment That Supports Well-Being in Old Age

**DOI:** 10.3389/fpubh.2020.589371

**Published:** 2021-02-04

**Authors:** Outi Hannele Jolanki

**Affiliations:** ^1^Gerontology Research Center, Faculty of Social Sciences, University of Tampere, Tampere, Finland; ^2^Department of Social Sciences and Philosophy, Faculty of Humanities and Social Sciences, University of Jyväskylä, Jyväskylä, Finland

**Keywords:** accessibility, senior housing, social environment, physical environment, aging in place, communal housing, well-being, qualitative analysis

## Abstract

**Background:** In different parts of the world new models of senior housing have rapidly appeared, which indicates that existing housing and care models are not fulfilling the hopes and needs of current and new generations of older people.

**Material and Methods:** This qualitative study focuses on one type of communal senior housing complex located in a mid-sized town in Central Finland. The complex was designed to have accessible low-maintenance apartments and common spaces, and to be near easily accessible green spaces, amenities, services, and public transport. The complex has a part-time community coordinator. The minimum age limit is set at 55 years. The data consists of 36 qualitative interviews with residents (21 women, 15 men) aged 66–93, conducted between November 2018 and February 2019. The semi-structured interviews were recorded and transcribed. The data analysis focused on how different aspects of the manmade, natural, and social environment were portrayed in residents' descriptions of day-to-day life. Theoretical framework adopted for the study draws from the ideas of environmental and geographic gerontology. The data was analyzed using positioning analysis which is one form of discourse analysis.

**Results:** The senior housing in this study fulfilled its promise of providing accessible a physical and social environment which encourages and enables residents to be physically active and independent, yet which also provides social activities and feeling safe. In this respect, the senior housing complex offered an environment which supports well-being and healthy aging. However, the residents' interpretations of what the senior housing complex represented varied. For some of the residents it was first and foremost a social place, which provided opportunities for social contacts and social activities. For some of the residents the most important were maintenance-free apartments and outdoor areas. The question remains as to how social practices, in the form of government policies and market systems can support the development of different kinds of senior housing which are affordable and accessible for all.

## Introduction

This study looks at new senior housing models, which are distinct from residential care homes, and aims to answer the research question of how this form of housing can offer the kind of environment that supports the health and well-being of older people. There are now many kinds of senior housing in different parts of the world, but irrespective of cultural and social differences, all these new models share the same goal, that is, to support aging in place and independent living. Some of the models also aim to increase reciprocity and mutual help at the same time. Before I look at the data produced by this study and the data analysis, I will therefore first look more closely at the policy goal of “aging in place” and how it relates to the development of new models for senior housing.

Aging in place (AiP) has become a policy goal around the world. The aim of AiP is to ensure that older people can continue living in their homes and familiar neighborhood without having to move due to a health problems and care needs (1–5). According to the World Health Organization (WHO) it means that by providing appropriate services and assistance it is possible to support older people's desire and ability to maintain relatively independent living arrangements within the community either in their current home or one that is more suitable to their current life situation (2004, p. 9). From this perspective the quality of housing stock, its physical characteristics and accessibility, and its transportation links to a wider community, are all important factors that support aging in place (1). Netherland et al. (6), for instance, argue that neighborhood and community are a crucial part of aging successfully—creating either barriers or supporting resilience and well-being in later life; while Lehning (7) links aging in place to age-friendly community initiatives, that pay attention to a multitude of non-human factors and services within local communities which could support independent living and encourage them to engage with the community. Age-friendly community initiatives vary depending on the policies adopted by local government in different places. It is for this reason why studies (such as the present one) which look at local initiatives in a particular cultural and regional context are important, as they can provide examples which, as Lehning (7) points out, researchers and practitioners can learn from.

The quality and extent of social relationships and participation in social activities affects the well-being and quality of life for elderly people (8–10). The availability of social support, social networks, and social activities can also affect health and functional ability (11, 12). The physical environment and geographical features of one's domestic surroundings can either enable or hinder physical activity. Since physical activity is not only linked to independent mobility, but to a general sense of autonomy and having some control over one's daily life, studies of older people's health and well-being need to look at the physical realities of their living environments (13, 14).

While there is often ambivalence about moving house in later life and relocation decisions arise from a complex set of reasons (15), some older people prefer relocation to staying put in an environment which no longer supports their well-being and accustomed lifestyle (2, 16, 17). The rise of these new models of senior or “in-between” housing (as they are also known), has appeared to fill the gap between ordinary private dwellings and residential care. As such, they are not residential care units or a form of service housing *per se*, although residential care is sometimes available or there may be a separate care unit in the premises. Senior housing models range from a purpose-built retirement villages, senior co-housing, virtual “Village model” to multigenerational co-housing and collaborative senior housing (9, 17–26). These models vary in respect to how they are funded, the age of residents', tenure type, and the role of residents in managing administration and maintenance of the building. What all these models have in common, however, is that they aim to support independent living as much as possible in spite of age-related health problems (26), while at the same time offering social activities, community participation, and mutual support and help (26).

There is some research which shows that residents have a mostly positive view of these new models of senior housing. They appear to increase subjective well-being by allowing the residents to feel that they have some control over the type of environment they live in (21, 23, 25); and they seem to provide new social contacts which not only enable the residents to continue lifelong social activities but even come up with new ones (17, 19, 24, 25, 27). Social contacts are particularly important since they decrease loneliness and increase feelings of safety and security (18, 21). A central concern is whether they are available for older people with poor health and memory problems, or for those with low-income (24, 28). While new senior housing models certainly seem to promote the well-being of residents, they are often an expensive solution that are mainly available only in urban areas in larger cities (18, 20, 24).

In sum, these studies give reason to think that new senior housing options which aim to provide accessible apartments and environments, have amenities and services within walking distance if not on the premises themselves, and offer opportunities for social activities, may provide an environment which promotes the residents' well-being in many ways. The present study analyzed in detail certain elements of senior housing that are often taken for granted, to see if the accommodation in question fulfills its promise of being a living environment that truly supports the well-being of its residents. The senior housing complex looked at here has owner-occupied, social rental housing, and “right of occupancy” housing (*asumisoikeus-asunto* as it is known in Finland and covered in more detail below), which sets it apart from the models featured in previous studies.

The present study looks at one type of senior housing located in a mid-sized town in Central Finland. In Finland majority of older people live independently in private owner-occupied detached houses or apartments. A number of people living in assisted living facilities is low, and for example of people aged 75 or older about 90% live in ordinary private houses or apartments. Many older people live alone and the trend is strengthening (29). In general about 40% of people aged 65 and older live alone, but in urban areas the percentages are even higher with more than 60% living alone. Older people often live in old and inaccessible detached houses or apartment blocks (29), but many plan to move to live closer to services and in apartment blocks. In short, there is clearly need for senior housing which is accessible, affordable, and offers appropriate amenities.

While this is a specifically Nordic context, it is based on the same ideas as many other senior housing models around the world. The complex was designed to have accessible low-maintenance apartments, lots of common spaces where residents could mix, and to be near easily accessible green spaces, amenities, services, and public transport. The aim of this architecture and design is to support an independent lifestyle, but also to facilitate contacts between those living there [cf. (30, 31)]. The residents all belong to a committee chaired by one of their own which meets every month to plan social activities (none of which are compulsory) and to convey the residents' views to the housing association in charge of administration. The block is thus a form of “communal” senior housing, but it is not co-housing since the block has a part-time community coordinator who organizes the activities together with the residents, none of whom are obliged to participate.

The senior housing model studied here is a “hybrid” in many ways: it is located in a semi-urban area close to services and public transport, but with good access to parks and forest areas. The complex consists of three high-rise apartment buildings which are linked on the first floor by hobby rooms, a laundry room, a lounge with kitchen area, and a restaurant. The complex has a mix of private owner-occupied, social rental housing and state-regulated “right of occupancy” housing which means that residents come from a fairly wide cross-section of socioeconomic backgrounds. Right of occupancy housing means that residents pay for only a small percentage of the value of the property plus a monthly fee which gives them a more secure right to live there than in normal rented accommodation. In fact, residents in this kind of housing have the same rights as those who bought their property privately—only more cheaply. The state helps with building right of occupancy housing, but it sets limits to the building costs and monthly fees that residents must pay. As well as the housing available for fully independent residents, a private care company provides 24/7 residential care in one part of the senior housing complex that they have rented out. The minimum age limit in the whole complex is set at 55 years, and at the time of the data collection the three apartment buildings had a total of almost 200 residents.

### Research Questions

Our main research question asked what the most meaningful elements were in residents' descriptions of the senior housing complex as a place to live, and more specifically what the elements were that they described most meaningful in making the decision to move there. The aim of the analysis was to examine what the man-made, natural, and social environment meant for the residents in their day-to-day life, and to answer the question of whether this type of senior housing met their expectations and has allowed them to age well. In the following we will show the results of the data analysis. Data extracts have been chosen which not only demonstrate shared and recurrent patterns of positioning but also the less common ways of portraying features of living in the senior housing complex.

### Theoretical Background

The concept of healthy aging is relatively complex, but for the study at hand we begin from the definition that for an individual person “healthy aging means having a sense of well-being, the capacity for independent activity, meaningful involvement, supportive environments, and positive attitudes. Being healthy is seen as having the resources for an everyday life that is satisfying to self and others” [(32), p. 101]. The fit between an individual person's capabilities and aspirations and his or her environment is thus essential in healthy aging (33). This study looks at the linkages between healthy aging and housing which means that the analysis needs to look at the physical, social, and mental aspects of health and well-being (34). A healthy environment refers to the natural, manmade, and social environment which supports the physical and social well-being of residents in a community. Housing is indirectly linked to well-being, for example, when one chooses to move to accommodation which is expected to be easily accessible and provide social support. It is directly linked to well-being, however, in terms of its physical and economic aspects—the size and quality of apartments, for instance, and housing tenure type (35). An important point to remember is that a precondition for choosing to relocate is to actually have some alternatives from which to choose. Following Clapham (36) it can thus be argued that housing policy is an important tool for improving the well-being of elderly people, insofar as it can create accessible and affordable housing options which provide a supportive living environment.

In age studies, geographical gerontology has drawn attention to the importance of studying how places affect the well-being of older people (37), but it also shows that with age, the meanings of places change (38). Wiles (39) has developed this notion further in noticing that places are processual and subject to ongoing negotiation; indeed, experiences and interpretations of place may differ, compete, or even conflict with one another. Furthermore, places are interrelated with other places on a different scale and at different times (39). Change is thus a fundamental part of this study, because it is looking at *relocation in later life* and living in a new environment, where people and places “co-constitute each other in an ongoing way through constant change” [(37), p. 218]. The senior housing complex is one form of communal housing and thus it aims to provide a sense of community and serve as *a community* for the residents. Yet, following Agnew's (40) distinction between *spaces* and *places* I point out that it is a matter of the data analysis to find out if the complex represents a community of belonging for the residents, or if it remains as a generic senior housing complex.

## Materials and Methods

The data consists of qualitative interviews with 40 residents (25 women, 15 men) aged 57–92. Majority of the interviewees were 70 years old or older (33) and majority of the interviewees lived alone (29). Of those living alone 6 were never married, 9 were divorced and 9 were widows. The interviewees housing tenure varied and 19 of them lived in right of occupancy housing, 8 in ordinary owner-occupied housing and 9 in rental housing. The semi-structured interviews were conducted between November 2018 and February 2019 and they were map-assisted, recorded, and then transcribed. At the time they were being interviewed, most of the residents had lived for about a year in the complex, and the interviews were held there, either in the residents' apartments or elsewhere on the premises (e.g., restaurant or hobby room). The authors interviewed them, so they also became acquainted with this living environment. Prior to the interviews they had also visited the senior housing complex in a group when they were introduced to how the place operated and to the actors involved in building and managing it.

In the data analysis, the focus was on how different aspects of the manmade, natural, and social environment were portrayed in residents' descriptions of day-to-day life, and, secondly on whether these portrayals give reason to argue that the living environment supports the residents' well-being. The focus of the study is thus on how the residents perceived housing and living environment as an element relevant for their well-being (34). To this end, concepts from positioning analysis (41) and environmental positioning analysis (42) were employed to analyze how residents describe themselves in relation to previous, current, and future living arrangements. Positioning analysis is one form of discourse analysis which focuses on language use to see if, and how, different features of the living environment are described as meaningful in daily life. This approach means that, irrespective of the research focus, the analyst has to remain open to each participant's own meaning-making activities. Following the theorization of geographical and environmental gerontology, we have used positioning analysis to study how the residents position themselves in relation to their current living environment. This approach brings with it a notion that the analysis has to look at both human and non-human dimensions of the environment, and how the residents position themselves and others as agents with the power to change their everyday life for better or for worse. As an example of positioning oneself in relation to other people is how the residents used the word “we” which sometimes referred to “we the residents” acting together (“we have the coffee time”), or alternatively to oneself and the spouse (“we like to go our own way”) as a dyad separate from the residents of the senior complex. The methodological application of environmental positioning proposed by Medeiros et al. (42) is particularly useful in unraveling different meanings of this particular type of senior housing which is founded on predefined ideas of accessibility and ideals of community living. In environmental positioning not just humans but non-human objects can be represented as actors, and the focus of attention is how the residents positioned themselves in relation to the objects. For example, one of the residents portrayed his previous living environment in relation to his health status (“when I got ill…I was left with a house that was too big and expensive”) and then the new environment that “drew” him in (“I could have moved somewhere else, but it's the sense of community here which drew me”).

In the analysis, the data was first organized by coding it with the NVivo software program, and then the coded text was subjected to discourse analysis to interpret the meanings of text segments within the context of the whole study. The final phase of the analysis was to draw together the results from this, and to interpret whether they confirmed that the senior housing complex provides a supportive environment for the residents. The coding consisted of going through transcribed interviews, systematically assigning a particular code to the language used in each case. The initial focus was on the physical (both man-made and natural) and social environment (in terms of services, social relationships, social activities). This provided the initial codes and sub-codes, but a more nuanced coding was created in the process of going through this data. The residents talked about the meaning of different dimensions of current living environment when they were asked about their reasons for moving, why they chose the housing complex over other choices, and when they were asked if they thought moving was a good decision. Occasionally current living environment was brought up in the context of other questions too.

## Results

When the residents talked about the life at the senior block two topics of concern came up in all the interviews, namely, the importance of being able to both have a choice and prepare for the future. Having a choice referred to two rather different things: namely, to relocation but also to everyday life in the housing complex. When talking about relocation, the residents highlighted that it was they who had made the choice to live in the complex; and when talking about everyday life in it, they emphasized the importance of being able to choose the extent to which they engaged in social activities. In this sense all of the residents interviewed positioned themselves as agents who had made a conscious decision to leave their previous home and relocate to a new “communal” living environment, but one in which they were able to choose their level of engagement in social activities. Relocation and a life in the senior complex thus provided a sense of being at least into a certain extent in control of one's life (32).

Environmental positioning entails also relational aspects between past, present and anticipated future (42). The residents often compared the suitability of their previous and current living environment for older people. They positioned senior housing complex as a suitable environment to grow old and a move was thus portrayed as an act to prepare for the future. In this context, future meant “aging”—in terms of its adverse effects—and relocation was portrayed as means to find a supportive environment to help cope with these. In short, residents exercised their agency in choosing to relocate to an environment that would support older people [cf. (37)]. However, in their interviews, residents anticipated the possibility of becoming less physically and cognitively able in the future. This view of the future restricted the range of places that they imagined to be appropriate for them to live in [cf. (40)]. So, when the residents described themselves as aging people, they were portraying old age as a kind of external force which restricted their agency in choosing a place to live.

### Physical Environment Enables Physical Activity and Social Encounters

The analysis examined the meanings to residents of various aspects of the physical and social environment in and around the senior housing complex—in terms of how these affected their decision to move there and find the new life there satisfying. Residents emphasized different aspects according to their own background, previous experiences, and housing history. For some, the design of the building and the surrounding physical environment made it easier to maintain their accustomed lifestyle. The following extract illustrates the importance to these residents of having the opportunity to continue certain physical activities and maintain social contacts. In the data extracts, I stands for the interviewer. The square brackets […] mark text that has been omitted for reasons of confidentiality, or in some cases to abbreviate the text to make it more readable. Without brackets three dots mark either a pause or interruption in the speech itself. Occasionally some information details have also been anonymized and marked in square brackets too [the neighborhood].

Extract 1. Anneli, female, living alone

*I*: OK, yep, so what was it like, what were your reasons for deciding to move here?

*Anneli*: Well, both my daughters live in [the neighborhood] with their families. And I decided to move here too when I retired [years] ago, because I had made the decision to no longer drive the car in wintertime. Sometimes it used to take an hour or so to drive here from [previous home]… So, I gave the car up altogether last autumn, and now I just walk from here as they live within a kilometer from here.

*I*: Right… so, in other words, it seems to have been a good solution then?

*Anneli*: Yes, it has been. Then there's also the fact that, throughout my life, I've always been involved in all kinds of organizations and associations, and have had a lot of activities. As it is, from here, I can manage very well, since there are six buses to the city every hour. And the bike—I've been biking so much this summer that I don't think I've ever biked as much in all the time I've lived in this city as I do now. Even though there's that huge [delay], I just went the other way around the lake. The road there is nice and level, you can get here just as quickly, there are no uphills, going uphill tends to be difficult at this age.

For some of the residents the location of the senior housing complex was important since they already had connections to the area either because children or other family members were living nearby, or because they had previously lived there themselves. In Anneli's case, the overall accessibility of the environment—in terms of location and local transport—not only made it easier to see her children and ensure that she could continue actively participating in various different organizations, but it also encouraged her to be more physically active overall—with all the cycling she was now doing. Even if they were not explicitly talking about it, residents hinted that age was an important factor in not just making the decision to move, but also in their day-to-day decisions. Anneli, for instance, describes herself as an active person who likes to walk, cycle and take part in different social activities on a daily basis, but nevertheless has some problems with mobility due to her age; and yet, for her, these problems were adequately addressed in her new living environment. Similar positive points about the environment were raised by other residents, when they described it in terms of other residents with more severe health and mobility problems, as we shall see in the next extract.

Extract 2. Martti ja Laura, a couple

*I*: So, we're interested in what exactly makes this kind of housing good, the factors that influence people to make the decision to move there…

*Laura*: OK

*I*: …and we want to find out more about the day-to-day life in it.

*Martti*: Well about that, yes, the environment is excellent for your average decrepit person, you know, for people who are not in good health.

*Laura*: Yes, they've got these excellent exercise…

*Martti*: Areas nearby.

*Laura*: …Yes, exercise areas that the council has built, which—when they were planning this place [the complex]—were going to be up on that hill, but then they brought the equipment down here instead…

*Martti*: To the lakeside

*Laura*: …by the lake, as people in the complex walk along the lakeside path quite a lot. And they don't—most of those with wheeled walkers anyway—don't have the strength to climb that hill.

In this extract Laura and Martti have highlighted the accessibility of certain outdoor facilities for frailer residents who otherwise have difficulty getting around. An interesting feature which crops up time and again in the data is that residents might occasionally portray themselves as old and having various health issues, yet will still draw attention to those in a worse physical condition to themselves. In this extract, for instance, Martti describes himself and his wife as being a bit “decrepit,” but he is quick to point out that they are nevertheless *not* one of the folk using wheeled walkers. In Laura and Matti's eyes, the outdoor areas serve the needs of older people with a range of varying abilities. One actor in implementing this is the council, which has equipped the park nearby with the kind of equipment which will make it easier for people living in the area to exercise more often.

As stated earlier, the housing complex was designed as a communal environment that encourages residents to socialize. However, the data analysis showed that for some, there were other aspects that were far more important in making the decision to move and their everyday life. The extract below comes from a part in the conversation where the interviewees—a couple called Seppo and Inkeri—had just stated they did not take part in the communal activities.

Extract 3. Seppo and Inkeri, a couple

*I*: OK, so are you saying you just haven't gone that much or what exactly?

*Seppo*: No, we haven't really gone. I'm not so keen on the poetry circle and the [unclear] circle… [laughter]

*Inkeri*: We, well we're more the kind of people, we like to go our own way. So, when it's the morning, yes it's usually before noon, we might… well, we usually go for a good walk—it's four or sometimes even five kilometers. There are good opportunities for walking round here.

*Seppo*: That's right, and everything is nearby too—except for that mall which was burnt down, but there is another [grocery store] near here, then there's [another store] less than a kilometer away and there are [other stores], so there are all these services, and this building is really good since it's new house and purpose-built for the old people […] Everyone must be over 55 years old. So, all of these things have been taken into account.

[Inkeri mutters something incomprehensible at this point]

*Seppo*: Yes, of course there is.

*I*: You mean outside or indoors?

*Seppo*: Yeah, there's a gym and everything.

From this extract, it is clear that it is vitally important for this couple to have easy access to outdoor walking areas, as well as amenities and services both inside and outside the complex. The couple portray themselves as independent-minded people who “go their own way” and appreciate their age-specific living environment without pursuing shared communal activities.

The following extract is an example of how a resident's previous living environment (whether an apartment or house), their life history, their health, and financial issues all affect their views of living in the senior housing complex.

Extract 4. Hannu, male, living alone

*I*: So what made you move here then? This is marketed as a communal senior block, but what really was it then—the central factor as it were—that made you choose this place?

*Hannu*: Well, these problems with mobility mainly. I mean, before this I was living in a two-story house, and with this mobility… these mobility problems, it just wasn't practical anymore. And it was quite hilly around there; hills—well there are some hills here too—but even small hills can actually cause quite a problem when moving around in this [assistive device]. And yeah, [the house] was unnecessarily big […]. Then, when I got ill [and personal circumstances changed] I was left with a house that was too big and expensive. And of course, there are these services here which also tempted me […]. I could have moved somewhere else, but it's the sense of community here which drew me …and the gym and restaurant were also important factors.

*I*: Right, so it was both the services and this sense of community which drew you. So, what does it mean exactly for you—this sense of community?

*Hannu*: Well, I guess it means at the very least, saying hello to other people [laughs] when you see them. And being able to bump into each other […] in the communal areas where you can chat if you feel like it […] I've never really got fully into that, but still, it's nice […] that there's this community, it provides some form of security.

As the above extract shows, for some people, the senior housing complex has been a place that makes life easier for those who have problems with their health and mobility. The fact that it has accessible indoor and outdoor areas, basic services like a restaurant and gym, and smaller and cheaper accommodation all weighed heavily in favor of moving there. Hannu's account also shows how having the chance to interact with others on a casual everyday level can also be important to those people who are otherwise not involved in the more organized social activities, so there are opportunities for residents to choose their level of social contact in the community. The data analysis showed, as we see in Hannu's case, that financial issues were an important factor in finding a smaller and cheaper place to live, but they also had an effect on how daily life was experienced in the senior housing complex. The following extract shows how, for some people, the shared spaces and shared facilities were important not just because of their accessibility, but also for financial reasons.

### Senior Housing as a Social Environment

The data analysis showed that while the physical and social environments were of equal importance and intertwined in residents' accounts, for some the social environment was more prominent in their accounts of day-to-day life. Social environment refers here to social contacts and activities provided by other people but also services. Services are made possible by decisions and actions of human actors and they entail activities of humans as service providers and users, and thus services are included in social environment in the analysis. In the following extracts, different aspects of social environment that relate to the well-being of residents will be addressed.

Extract 5. Raimo, male, living alone

*I*: OK, so if we were going to sum it all up, what then are the good sides and the bad sides here?

*Raimo*: Well, this building is very good, it's so quiet and, well, everything seems to be working. And if you want some company, there's a large shared living room so at a certain period of the day there are people there—not all the time, but anyway—you can sit and watch the big televisions they have there with other people if you want, and be a couch potato.

*I*: OK, so do you usually go there?

*Raimo*: Well, um, yes, I go there every now and then, and then there's coffee, we have a “coffee time” and a system where you can buy a cup of coffee […] that costs 50 cents. So, once we've set it up, we can buy our own coffee and so on.

[…]

*Raimo*: And then, then we have the gyms, they are free, and laundry is free, and then there's the sauna and that's free too—you just book the time you want to go in advance.

*I*: Right. So, do you book a time?

*Raimo*: I have my own time, a time that I go. […] Yes, I have my own [sauna] slot.

The extract above is a good example of those interviews where residents highlighted the advantages of the purpose-built facilities and the overall peacefulness of the living environment. It is also representative of those residents did not take a very active part in community life, and yet described some of the shared premises as a continuation of their own apartment. For some, these common spaces clearly allow residents to engage in activities as an organizer, while for others, like Raimo, it's clearly enough to socialize with people as just an observer.

Like Hannu, Raimo seems to view social activities as something you can choose to do from time to time, but only if you feel like it. He describes how watching TV in the living room gives him the chance to be in a group, albeit as a passive “couch potato.” In addition, participating in making and sharing the daily coffee provides an opportunity to be part of social group without there being any more specific activity going on. In this way, Raimo sees himself as a member of the community, one of the “us” involved with organizing activities without feeling that he is the sole or primary organizer. In Finnish culture, an afternoon coffee-break (which usually takes place between 1 and 2 p.m.) is treated as an important part of the day. If you take afternoon coffee with others, it shows that you are at some level willing to engage with the community and socialize with them.

Raimo's extract also draws attention to the important aspect of financial issues. One of the apartment houses in the complex had people living on rental in social housing, or in right of occupancy housing which is considerably cheaper than non-subsidized private housing. Even though financial issues did not come up explicitly in linkage to living environment i.e., nobody positioned themselves as poor or talked about financial issues as a reason to choose this type of living environment financial issues were not unimportant. The free laundry, gym and sauna, as well the availability of a big TV and cheap coffee were mentioned as such resources are not ordinarily available in most apartment buildings, and going to a public gym or sauna is likely to be too expensive for some of the residents. It is also worth mentioning here that sauna, like afternoon coffee, is another very important part of Finnish culture, and each of the complex's three saunas were frequently mentioned by interviewees as being an important part of day-to-day life. The senior housing complex thus provides two symbolically important features of Finnish culture—afternoon coffee and sauna.

While some of the residents, as we have seen, preferred to see themselves more as passive observers in organized events and activities, for others it was a place where they could engage in their personal interests and put their skills to use by creating a program and events for the whole housing complex.

Extract 6. Timo, male, lives alone

*I*: So that's what you're involved in (residents committee); but what about these coffee breaks and so on, how are you involved in those?

*Timo*: Yes, all the time […] There's a good […] restaurant. There are good common areas where we can mingle too, and then there's a gym and everything. There's a barber's too—even a chiropodist. You don't really need to leave here except to shop.

*I*: So do you make use of them then, these other services?

*Timo*: Yeah, I do use them, I do […]. Yes, I use the barber and the chiropodist and I sometimes go to the gym. And let's see, yes, we have this regular discussion group, and then there is singing. There's like a singing evening, in the neighboring apartment block they have choir singers, and I'm pretty good at too, I'm quite good at singing. So, there's two of us [laughter], who organize the singing evening [laughter].

This is a good example of how residents might highlight quite different aspects of day-to-day life that make them feel good about their living environment. In Timo's case above, it's not just the availability of services and amenities in his living environment that is important, but also the social relationships and activities which take place there. Some residents described the senior block as a place that enabled the residents to bring their own special talents to the social life of the community. In Timo's case it is singing—he portrays himself as a “good singer” and as active in organizing the singing evening. But this is not all; in describing himself as an active member of the community—in the residents committee and as an active user of the services available on site—Timo uses the first person plural. He sees himself clearly as part of a group, a “we” who attend social groups and events together. For some residents like Timo, therefore, the senior housing complex represents a place which can, in many ways, offer “everything.”

In the next extract, the perspective is again slightly different, but the interviewee again underlines the importance of services and social contacts.

Extract 7. Rauha, female, living alone

*Rauha*: [talking about her previous apartment] So it would have been possible to live there during the renovation, but I thought it'd be better not to have to move twice. And then the second thing was to do with how I was going to spend the rest of my life, since you don't have to be alone here. You can go into the living room and there's always, there are always people there, so the loneliness is not so bad, and when you want to be left alone, you can do that too. So there's nothing… well, it's basically safe here. And then there's the surroundings, with countryside and the lake nearby.

*I*: OK, so let's see, did you know about these things before you moved here, and was that what interested you about moving here…this sense of community?

*Rauha*: Yes, yes, that was exactly it.

*I*: So, it was not just the apartment?

*Rauha*: No, not really, it was the thing that, well I was thinking I'm still fit enough not to have to go to an actual care home, so this was a sort of in-between option, and there are all kinds of good things about this place. Yes, and if your health deteriorates, then there is [private health care provider]… as long as you have enough money, [laughter] you can move there.

*I*: Yes, that's right-

*Rauha*: So, if you look at it holistically, it's the idea of living as independently as you can for as long as possible, and there are services here too…practically everything you need.

In the above extract, Rauha is describing her living environment in terms of how she sees the rest of her life. Aging and old age were often cited by interviewees as a kind of “outer force” which dictated certain necessary criteria for future accommodation. For many, the senior housing complex fitted the bill insofar as it provides a buffer against social isolation and loneliness in old age, and some form of support should one's physical health require it. Not all residents saw themselves as prone to loneliness or social isolation, but it was more something that they feared might happen if they continued living in ordinary accommodation. Moving to the kind of accommodation they were now in was therefore a preemptive way to tackle this possibility, and to improve one's life in old age. Another crucial factor raised by many residents, and in Rauha, Hannu and Raimo's accounts above, was the matter of not only being able to choose one's social activities but also one's level of engagement in them. Senior housing should offer the possibility of company if desired, so that being alone or with others is a matter of choice rather than obligation.

As well as providing the chance to socialize, another important feature of the social environment raised by residents was that it should have easily accessible services and amenities. In the interviews, one of the most frequently cited of these services was public transport—either the local buses with a stop nearby, or dial-a-ride ones which would pick up and drop off residents right outside their home. In the following, Rauha talks more about these in relation to her own mobility issues.

Extract 8. Rauha

*Rauha*: I can't really add anything else to that except to say that this is an ideal place since services are close, so you don't really need to go anywhere, but at the nearby mall there is everything anyway.

*I*: OK

*Rauha*: And there are buses too, then there's also this [name of dial-a-ride service] minibus, which picks you up and drops you off in front of this building […].

*I*: Do you use it?

*Rauha*: Yes, yes, yes, whenever I need to get out, it's so handy since you don't need to walk anywhere. But I do also use the [ordinary] bus quite a lot too, as it's not like I need it [dial-a-ride], but for someone like me with mobility problems, and I do have a [wheeled walker] since my back hurts so much [it's nice]. I sometimes need to sit down when there are no seats around, so that's why I use one, but otherwise I don't need one.

In the majority of interviews, public transport was seen as a crucial resource, especially among those who did not have a car, but it was also important for those that did, who were thinking about a future when they might not. For many, like Rauha, both kinds of bus service allowed her to run errands independently which, if there are no friends or family nearby to assist, is clearly an advantage. For those residents who were single or childless, public transport was also essential for shopping elsewhere. It is interesting here, that Rauha downplays her need for the dial-a-ride option by pointing out that she also uses the normal bus—dial-a-ride is only for special occasions.

One thing this senior housing complex has that does not seem to be available in most other senior housing contexts is a community coordinator. This coordinator works part-time on site, and their salary is paid out of residents' monthly fees. The senior housing complex also has a private care provider who operates a small care home within the premises. Both of these were often mentioned as welcome reassurance of support in the future, even if they weren't services that were currently being used.

Extract 9. Martti ja Laura, a couple (see also extract 2)

*Martti*: Yes, but I was talking about the community coordinator; she can help with things like if you can't get your internet banking, for instance, to work on your computer. If she can't help, then she will at least look for someone who can…

*Laura*: And she's organized all sorts of things in our common room.

*Martti*: And if you have papers you need to sort out with the bank, then she'll help you with that.

[…]

*Laura*: So yes, it really is excellent, and now there is also this [private service provider] here so, and I'm thinking of our situation if we get a bit more worse for wear, then it's just a case of ticking a few more boxes on our agreement contracts to have those services included. There are some people here who really have trouble starting their day so, even now, the people at [private service provider] take them breakfast […], delivered directly to their home, and…

*Martti*: And they make sure they take the right doses of medicine.

*Laura*: Yes… all these kind of things

*Martti*: They get the medicine from the pharmacist.

Laura: So then it's quite natural […] at this stage, when you cannot, when you start to lose your marbles, or there is some other reason that you need […] round-the-clock care, that you can just be transferred there.

*Martti*: And the [private service provider] gives you these safety alarm […] wristbands.

*Laura*: And for those of us who live here, we get a different price for this service…

*Martti*: …for the continuous supervision.

[…]

*Martti*: And, what's really excellent compared to [previous home location] is the, well, a kind of, safe environment here. Of course, we had neighbors there, and we kind of got on OK, but here there is a broader network of people that give you this greater feeling of safety.

In many of the interviews, the community coordinator cropped up as the person who sorts out residents' various practical problems, especially with the internet, computers, banking or other official business. Another important job for the community coordinator mentioned in the interviews was arranging a range of activities for the residents. In this respect, the coordinator eased the burden of responsibility on those residents who were trying to organize activities, so residents felt encouraged to not only continue organizing activities, but also to engage in a wider range of them than might have been possible without the involvement of the coordinator.

The private care service provider also cropped up in the interviews as a form of support that might be relied on in the future should the need arise; it was thus seen as another potential resource that contributed toward making the living environment feel safer. As we could see in Martti's and Laura's interview above, knowing that the private care was in the same housing complex made them feel that it was quite safe to continue living in individual apartments, as relocation could be done gradually with an intermediate level of care being brought to them in their apartments, for instance, as one level of service.

In the last part of the extract above, Martti compares the “safe environment” of his present home with how he felt in his previous home. This was a theme often brought up by residents in their interviews. This feeling “safe” was expressed in a range of ways depending on what they were previously accustomed to: from Hannu's feeling that residents were able to just say “hi” to each other, to Martti's feeling that residents knew each other a lot better in the senior housing complex than in an ordinary apartment block; while for others, the feelings of safety were linked to the accessibility of amenities and services nearby and on the premises. Another important factor that increased these feelings of safety was knowing that there were resources provided for onsite which residents would be able to benefit from if their mental or physical health deteriorated with age. In most cases, residents had cited this eventuality as being one major reason for wanting to move into senior housing in the first place.

## Discussion

The senior housing in this study fulfilled its promise of providing a good example of a physical and social environment which encourages residents to be independent, enables them to continue life in the manner they've been accustomed to, yet which—at the same time—provides a new social network and activities that they can take up should they so choose. In this respect, the senior housing complex offered an environment which supports well-being and healthy aging (32). However, the analysis showed that the residents' interpretations of what the senior housing complex represented varied to such an extent that it was clearly not the same place for them all [cf. (39)]. Our study adds to the previous studies of collaborative housing by showing that the residents in collaborative senior housing may position themselves in relation to other residents but equally often to non-human aspects of the housing complex. While some residents value communal activities for some the primary value of housing lives elsewhere. Since the complex was advertised as a form of *communal* senior housing, there must certainly have been expectations that it would offer more than regular senior housing. Nevertheless, for some it was just that; a physical space, which via accessible and maintenance-free apartments and outdoor areas enabled them to continue their lifestyle and maintain existing social networks and family relations. Common areas and activities were described as being a possibility should they so choose, but not relevant to their own daily life. For others, however, the senior housing complex was first and foremost a social place. The senior housing complex was also portrayed as *a community in the making*—offering opportunities for new social contacts and social activities. These different perspectives were clearly illustrated in the kinds of language used by interviewees. While some residents emphasized that their main social contacts and social life was outside the housing complex, some of the residents talked about *our* common areas and the *we* who would meet, greet, and organize activities together. This use of the first-person plural clearly shows that they positioned themselves as members of a community to which they felt a sense of belonging.

An important novel finding of this study was received by using Agnew's (40) distinction to places and spaces. The senior housing complex was a special *place* for some residents and they felt they were very much part of it. At the same time, there were those who kept their distance and participated either very little or not at all in communal activities. For these residents, the housing complex was a generic living environment suitable for older people; *a space* which served its purpose but could be swapped with any other senior housing. This does not mean that attempts to create communal senior housing which encourages social contact is futile, but is simply a reminder of the fact that seniors are as heterogenous a group as any other in our society, and the people in this group have their own interests, preferences and aims in life. The residents chose to relocate to an environment which they anticipated as being supportive for older people, but they also chose the level of participation in activities within the housing complex. This reminds us that older people are agents in their environments (37), and not all of them want to grow old in old homes but are keen to actively shape their own living environment in later life (40).

Creating a living community is a process (39), and aging changes how we see our living environment, our homes, and the places we live (38). Residents spoke about the future in terms of anticipating deteriorating health and the restrictions this would cause. These “restrictions” determined views of appropriate housing and living environments for older people [cf. (40)]. A common feature in the data was that residents described themselves as aging people, and in so doing, old age was portrayed as an external force which set limits on their agency over determining places to live.

While there were clearly differences in regard to the importance of the communal aspects of the senior housing complex, there was general unanimity about the importance of its physical location: the easy access to amenities and services, the pleasant natural surroundings with the nearby lake and forest paths, and the good public transport. This accessibility also supported physical and social activities seen to be crucial for promoting well-being, as shown in previous studies (43). The accessibility of the physical environment and services allowed for greater mobility, which made it easier for residents (including those with health and mobility problems) to feel they had more control over their day-to-day life [cf. (13, 14)]. Special services—such as the community coordinator and dial-a-ride bus—were other important local initiatives which interestingly added to a sense of simultaneously feeling safe yet also independent. Human and non-human actors and policy practices clearly had a meaning in enhancing the well-being of the residents too, as stated in other studies (1, 7). New feature in some models of senior housing is to have ordinary and service housing within the same complex. In this case, novel finding of the study is that the presence of a private care company and 24/7 care unit on site were seen as a potential resource for the future rather than important on a day-to-day level. Thus, their meaning was more symbolic than practical, but potentially important in the future and adding to a sense of safety. This result shows that integrating service housing units to ordinary senior housing can be an important feature adding to the well-being of the residents.

For many residents, the physical and social environments were intertwined in many ways. The shared first floor, with its common room, restaurant, saunas, gyms and laundry facilitated contact between residents, so the architecture and design of the housing did encourage socializing [cf. (30, 31)]. For those more interested in participating in shared activities the communal senior housing offered a wide range of activities and social possibilities, as described in previous studies (17, 19, 27). A very important novel finding was that the residents' level of engagement in social activities varied from being simply observers to actively organizing events, but knowing that these activities—so meaningful to some residents—were simply there, was often enough to create a sense of belonging in the community, even among the more passive residents. Many residents talked about how the senior housing complex helped decrease feelings of loneliness, while increasing feelings of safety and security [cf. (8, 18, 21)]. Yet another novel finding was that even those who were not “deeply involved” in communal activities mentioned that simply knowing each other increased feelings of safety. The future life of the senior housing complex will prove whether it will have a lasting effect on residents' well-being and whether it will continue to function as a supportive community in the long run [cf. (22)]. For now, the communal senior housing complex, with its accessible environment and nearby amenities and services, offer a supportive environment that adds to the resources of its residents so that they can live a life that is satisfying both for themselves and for others.

The strength of the study is that the data represents views of people of different ages, both men and women, different housing tenures and with different reported health status. In addition, theories coming from geographical gerontology and environmental studies together with detailed analysis of language use and positioning analysis produced results that provide new ways to see the meaning of collaborative senior housing for the residents, as well the meaning and relation of human and non-human aspects in creating living environments that support well-being in later life. The limits of the study come from the fact that the data come from small-scale study that represents rather rare senior housing solution, and a small social and cultural context of one of the Nordic countries. The limits of the study mean that the results cannot be generalized directly to other countries or the analysis cannot be replicated as such. However, these limitations have been acknowledged and addressed and do not make futile the meaning and applicability of the study results.

## Conclusions

New models of senior housing have appeared to fill the gap of so called “in-between” housing. These new models have the potential to offer an age-friendly environment where independent living is possible even with an age-related deterioration in functional abilities. Many of the new housing models aim also to offer social activities, mutual support, and help. However, the residents interviewed here had a range of expectations concerning the housing, just as they acted differently from other residents in their day-to-day life. These concerns do not mean that we should give up on new communal senior housing models, but instead prove the importance of approaching them as processes, which can develop according to how residents interact with their physical and social environment (39).

The senior housing complex analyzed here was in a Nordic country that purportedly has a good level of public social and health care services, with home care and residential care which is supposed to be available for all those in need of them. And yet, the rapid increase in new senior housing models could be an indication that existing housing and care models are not fulfilling the hopes and needs of current and new generations of older people. Therefore, I argue that the model discussed here may work even better in countries with less existing services for older people, and which traditionally rely on private self-care, family care, or housing solutions. While the results of the small-scale qualitative study are not directly generalizable to other countries the results offer a point of comparison, and provide innovative model of senior housing that can be experimented elsewhere to develop senior housing which supports well-being of the residents.

Firstly, this analysis showed that this type of housing has the potential to provide a social environment that supports the health and well-being of older people. Secondly, the hybrid nature of this kind of senior housing—with accessible premises, shared resources, social activities, and an on-site care unit—can provide a socially and economically viable solution for senior housing. However, there are some questions and concerns that need to be addressed—this kind of senior housing is available in a number of different countries, but mainly for only those who already have the health and financial resources to find a new place to live (18, 28). Many such options are rather expensive and available for the most part in only urban areas (20, 24). The question therefore remains as to how social practices, in the form of government policies (on both a national and local level) and market systems can support the development of different kinds of senior housing which are affordable and accessible for all (36). Alternative solutions, like the one studied here, which combine an age-friendly living environment with a communal type of housing, and which also mix tenures, require collaboration between the private, public, and third sectors, not to mention the active participation of the residents themselves (perhaps the most important agents in finding these solutions). Hybrid models require flexibility and innovation from all the actors involved, but as this analysis has hopefully shown, they may well offer a living environment for older people that is truly worth aspiring to.

The rapid growth of different senior housing models signals the need for a variety of in-between housing options for older people, and housing policy is the key to deciding if these exist, and if so, it also decides their location and tenure. Following Clapham (36) it can thus be argued that housing policy is an important tool for improving the well-being of elderly people, insofar as it can create accessible and affordable housing options which provide a supportive living environment.

## Data Availability Statement

The datasets presented in this article are not readily available because the data is in Finnish language. The anonymized data will be made available 2025. Requests to access the datasets should be directed to outi.jolanki@tuni.fi.

## Ethics Statement

The studies involving human participants were reviewed and approved by Human Sciences Ethics Committee, University of Jyväskylä, Finland. The patients/participants provided their written informed consent to participate in this study. Written informed consent was obtained from the individual(s) for the publication of any potentially identifiable images or data included in this article.

## Author Contributions

OJ has participated in the research group in planning the research project on senior housing, designing the data collection, collecting the data and the administrative task of data preparation for analysis purposed. OJ has planned the study at hand, analyzed the data, and written the final text as well revised the manuscript.

## Conflict of Interest

The author declares that the research was conducted in the absence of any commercial or financial relationships that could be construed as a potential conflict of interest.
