# Supplementary material for: Senior Housing as a Living Environment That Supports Well-Being in Old Age
Source: Front Public Health. 2021 Feb 4;8:589371. doi: 10.3389/fpubh.2020.589371 (PMC7890193; doi:10.3389/fpubh.2020.589371)
Supplement: Supplementary file 1 [file Data_Sheet_1.PDF]

Senior housing interview guide

1) Life history and history of residence

Instructions: Map-assisted narration i.e. describing one's life history by way of places of residence and relocation using a map for assistance. The map can be used to discuss other topics too.

- Could you tell me where you were born?

    The interviewee's life story recounted in an informal fashion: place of birth, childhood, youth, adult life and retirement

    Check-list: schooling and education, employment history, family life (spouse/partner, children, other close ones) and living arrangements

2) Current situation

Housing situation

- When did you move here?
- Why did you decide to move?
- Why did you choose this place, and did you consider other options?
- In hindsight, was moving the right decision? Could you give an example of what is good/what works well?

Residential area and the neighbourhood

- What do you think of this neighbourhood as a place of living?
- Do you go for walks or take outdoor exercise here in this area? How often do you do it/Do you do it regularly?
- Do you use any services available in the area? For example, shops, post office, public transportation, library or other services?
- In your opinion, what are the best qualities of the neighbourhood? Are there any negative qualities?

Plans and dreams for the future

- Have you thought about moving to some other place in the future? Have you considered service housing as an alternative?
- In your opinion, what would be the best place to live in later life?

Close relationships and social networks in the neighbourhood

- Who are the members of your family? How often do you meet with them and how do you keep contact?
    - Where do your closest friends and family live?
- Do you see and meet with the other residents? Where and in which situations do you meet them?
- How about friends? How do you keep in contact with each other?

The quality of and satisfaction with social relationships

- Who are the most important people to you?
- Do you feel that you have people you could ask for help should you need it?

Need for assistance and support

Informal assistance

- What is your health like?
- Do you need help with some everyday matters? What kind of situations these are?
    - Where do you get help or who helps you? (family, friends, private or/and public services)
- Could you ask help from other residents here should you need it?
- Have you been in a situation where you have helped others? (for example, a family member, a neighbour, a friend or someone else)

Public and private services

- Do you use any public or private services? What kind of services?
- What are your experiences of the services?
- Is there something with which you would need help, but it is not available? What do you think is the reason you have not received sufficient assistance?

The concluding comments

- Is there something else you would like to say or is there any topic that has not been discussed?
